# Supplementary material for: Tip Lesion Most Frequent FSGS Variant Related to COVID-19 Vaccine: Two Case Reports and Literature Review
Source: Vaccines (Basel). 2024 Jan 8;12(1):62. doi: 10.3390/vaccines12010062 (PMC10821173; doi:10.3390/vaccines12010062)
Supplement: Supplementary file 1 [file vaccines-12-00062-s001.zip › vaccines-2701296-supplementary.pdf]

OPTICAL AND TRANSMISSION ELECTRON MICROSCOPY (TEM) OF PARAFFIN-EMBEDDED RENAL BIOPSY TISSUE

PROCESSING FOR OPTICAL MICROSCOPY

1. Fixation: Kidney tissues obtained by biopsy are fixed in 10% Carson's buffered formaldehyde for 2–24 hours.

2. The fixed material is processed in a 3-hour protocol using a Leica ASP 300S tissue processor.

3. The samples are embedded using a Leica Arcadia paraffin embedding station.

4. The embedded samples are sectioned using a Leica RM2255 automated microtome.

5. The samples are stained for optical microscopy.

USE OF PARAFFIN-EMBEDDED MATERIAL FOR TEM

1. The block that will be used for TEM is placed in the HistoCore Arcadia (Leica) at a controlled temperature of 60°C to soften the paraffin.

2. The material detached from the block is dewaxed using three xylene baths for 15 minutes each.

3. The dewaxed material is hydrated using descending concentrations of ethanol, with exposure to two solutions of absolute ethanol for 20 minutes each, one solution of 95% ethanol for 10 minutes, one solution of 70% ethanol for 10 minutes, and one solution of 50% ethanol for 10 minutes, followed by hydration in water. The hydrated material is processed for TEM as described next.

PROCESSING FOR TEM

1. The hydrated samples are exposed to 10% Carson's buffered formaldehyde for 12 hours.

2. Dissection: Each kidney tissue fragment is cut into smaller fragments of no more than 3 mm.

3. Washing: The fixative solution is discarded and each sample is washed three times for 10 minutes each time in 0.1 M cacodylate buffer, pH 7.2.

4. Post-fixation: Following the final wash, approximately 500 µl of 1% osmium tetroxide (OsO_4) is added to the 0.1 M cacodylate buffer and stirred for 1–2 hours, depending on the thickness of the sample.

5. Rinsing: Discard the OsO_4 and wash the samples three times for 10 minutes each time with deionized or distilled water.

6. En bloc staining: Following the final wash, add 500 µl of 2% uranyl acetate solution to the wash solution. Each sample is carefully removed from the surface of the stain solution without disturbing the precipitate and left in a fume hood for one hour at room temperature protected from light.

7. Rinsing: The uranyl acetate solution is discarded and each sample is washed three times for 10 minutes each using deionized water.

8. Dehydration: Samples are dehydrated by sequential exposures to 50% and 70% ethanol (10 minutes each), 95%, 100%, and 100% ethanol (15 minutes each), Acetone 1, and Acetone 2.

9. Impregnation: Following the acetone steps, each sample is left in a 1:1 acetone: epon resin mixture without catalyst for 30 minutes. This mixture is then discarded and replaced with a 1:2 acetone: epon resin mixture and stirred overnight at room temperature. The mixture is removed and replaced with epon resin without catalyst for 6–8 hours.

10. Polymerization: Each tissue block is transferred to a mold containing freshly prepared epon resin with catalyst and polymerized and left in a 60°C oven for 48 hours.
